# Survivin expression in patients with newly diagnosed nodal diffuse large B cell lymphoma (DLBCL)

**DOI:** 10.1007/s12032-012-0232-x

**Published:** 2012-04-13

**Authors:** O. Markovic, D. Marisavljevic, V. Cemerikic-Martinovic, T. Martinovic, B. Filipovic, D. Stanisavljevic, R. Živković, J. Hajder, N. Stanisavljevic, B. Mihaljevic

**Affiliations:** 1 KBC “Bežanijska Kosa”, Bezanijska Kosa bb, 11000 Belgrade, Serbia; 2 Beolab, Belgrade, Serbia; 3 Institute of Histology, Faculty of Medicine, University of Belgrade, Belgrade, Serbia; 4 Institute of Statistics, Clinical Center of Serbia, Belgrade, Serbia; 5 Institute of Hematology, Clinical Center of Serbia, Belgrade, Serbia; 6 Faculty of Medicine, University of Belgrade, Belgrade, Serbia

**Keywords:** Diffuse large B cell lymphoma, Apoptosis, Survivin, Immunohistochemistry, Prognosis

## Abstract

Survivin is one of the inhibitors of apoptosis proteins (IAP) that might play an important role in the pathogenesis of diffuse large B cell lymphoma (DLBCL). The present study was designed to investigate the clinical and prognostic significance of survivin expression in nodal DLBCL. We analyzed lymph node biopsy specimens obtained from 56 patients with newly diagnosed nodal DLBCL, treated with immunochemotherapy (R-CHOP). The expression of survivin was analyzed using the standard immunohistochemical method on formalin-fixed and routinely processed paraffin-embedded lymph node specimens and evaluated semiquantitatively as a percentage of tumor cells. Survivin immunoexpression (>45 % positive tumor cells) was found in 22 (39.28 %) and observed as cytoplasmic staining in 15 patients, or mixed (cytoplasmic and nuclear) staining in 7 patients. A significant difference in survivin immunoexpression was noticed between the GCB and the non-GCB subtypes of DLBCL (*p* = 0.031). However, survivin immunoexpression had no significant association with IPI, “bulky” disease, extranodal localization, hemoglobin, Ki-67 immunoexpression or other clinicopathological parameters. A univariate analysis showed that survivin positivity was an unfavorable factor for therapy response and a predictor of shorter survival in patients with DLBCL (*p* = 0.048 and *p* = 0.034, respectively). Patients with survivin overexpression experienced a relapse more often than patients without expression of this apoptotic protein (27.3 vs. 11.8 %), but this difference did not reach statistical significance (*p* = 0.131). The results of this study showed that disregulation of survivin expression had an important role in the determination of the course of the disease in patients with nodal DLBCL treated with R-CHOP. Therefore, survivin represents a potential target for therapeutic intervention in DLBCL.

## Introduction

Diagnosed nodal diffuse large B cell lymphoma (DLBCL) is an aggressive disease with variable clinical, histological, immunophenotypic and cytogenetic features [1]. Although the disease is very heterogeneous, initial treatment applied to all patients with this type of lymphoma is almost always the same [1], namely, in recent years, all newly diagnosed patients with DLBCL have been treated with R-CHOP protocol, which is the gold standard in treating this type of lymphoma [2]. Although the addition of rituximab contributed to the improvement of the therapeutic response and survival of patients with DLBCL, some patients do not achieve a favorable therapeutic response or relapse after successful treatment [2]. Thus, new prognostic tools for identifying the patients who will not experience remission following initial therapy and who need an additional or more aggressive therapy are needed.

Apoptosis is a genetically regulated cell death mechanism essential for the development and homeostasis of multicellular organisms. It is regulated by two families of proteins: the BCL2 family, comprising both pro- and anti-apoptotic members [3], and the inhibitor of apoptosis protein (IAP) family, consisting only of anti-apoptotic molecules [4]. To date, eight members of the IAP family have been identified in humans, among which survivin with 142 amino acid residues is the smallest. Expression of survivin is consistently associated with the inhibition of induced cell death in cell culture systems and in transgenic animals as well, whereas survivin suppression triggers caspase-dependent apoptosis both in vitro and in vivo [5]. Inactivation of survivin expression can restore TRAIL sensitivity in resistant non-Hodgkin lymphoma B cells [6].

Previous studies have shown that deregulation of apoptosis signaling cascade is an important factor in the pathogenesis of lymphoma and that such deregulation may be an important cause for chemotherapy resistance and a poor prognosis in DLBCL [7–13]. Most of these studies analyzed patients treated with conventional chemotherapy (CHOP) [9–12], but there is only one study that analyzed the prognostic significance of survivin in patients with DLBCL treated with immunochemotherapy (ICH) [13]. Therefore, we analyzed whether immunoexpression of anti-apoptotic protein survivin influences the therapy response and survival of patients with nodal DLBCL treated with immunochemotherapy.

## Patients and methods

### Patients

We analyzed 56 patients with de novo nodal DLBCL diagnosed from January 2004 to September 2008. The diagnosis was established according to the criteria of the World Health Organization classification [14]. A number of clinical variables were particularly analyzed: age, gender, clinical stage, ECOG, IPI, serum albumin, C-reactive protein, ß_2_-microglobulin, LDH, hemoglobin concentration and “bulky” disease. The staging of the disease was done according to the Ann Arbor classification [15]. The International Prognostic Index (IPI) score was determined, as described previously [16]. The patients were subdivided into the GBC and the non-GBC types according to the model proposed by Hans et al. [17]. Patients with human immunodeficiency virus positivity and patients with primary extranodal disease (CS IE or IIE) were excluded from the study.

This study complied with all the provisions of the Declaration of Helsinki and its current amendments and was conducted in accordance with the Good Clinical Practice Guidelines. The study was approved by the Institutional Ethical Committee.

### Treatment

All patients were treated with immunochemotherapy: 51 patients received the R-CHOP regimen consisting of cyclophosphamide, 750 mg/m^2^; doxorubicin, 50 mg/m^2^; vincristine, 1.4 mg/m^2^ (up to a maximum dose of 2 mg) on day 2; and prednisone, 60 mg, administered orally, on days 2–6. Rituximab was administrated at a dose of 375 mg/m^2^ on day 1. The treatment was repeated every 3 weeks. Five patients received the R-EPOCH regimen consisting of rituximab on day 1; etoposide, 50 mg/m on days 2–5; doxorubicin, 10 mg/m on days 2–5; vincristine, 0.4 mg/m^2^ on days 2–5, administered as a continuous i.v. infusion; prednisone, 60 mg/m^2^, administered orally on days 2–7; and cyclophosphamide, 750 mg/m^2^ on day 7. The patients in clinical stages II–IV were treated with six to eight cycles of immunochemotherapy. The patients in the first clinical stage were treated with three cycles of immunochemotherapy and “involved” field radiotherapy. The irradiation therapy (30–40 Gy) was applied after immunochemotherapy in the patients with “bulky” disease or with residual disease. Treatment response was evaluated according to the International Workshop Criteria [18].

### Immunohistochemical studies

A tumor tissue was obtained from every patient by lymph node biopsy, fixed in buffered formalin, at pH 7.4, and embedded in paraffin. Then, 3-μm-thick paraffin-embedded tissue samples were cut, deparaffinized in xylene and rehydrated in water. The lymph node specimens were analyzed by conventional light microscopy examination and immunohistochemical analysis. The immunoexpression of survivin was assayed by means of the avidin/biotin/peroxidase complex method (LSAB 2, DAKO or Ultravision LP Detection system, Labvision) using aminoethylcarbazole or DAB as a chromogen. A heat-induced epitope retrieval method was used before the immunostaining, namely sections were placed in 0.01 mmol/L citrate buffer at pH 6.0 and heated twice in a microwave oven for 10 min per cycle. The sections were stained with a survivin antibody (RB-9245-R7, Labvision, dilution 1:50). The antibody was incubated for half an hour at room temperature. After the development of the chromogen, all slides were counterstained with hematoxylin. The control sections were immunostained under identical conditions, substituting the primary antibody with a buffer solution. The tissue of prostate carcinoma served as a positive control. The expression of survivin was evaluated semiquantitatively as a percentage of positive cells of all tumor cells. Only cells three times larger than small lymphocytes were analyzed. At least 500 cells were counted in each case. Lymph node samples were evaluated at 100× and 400× magnifications and independently analyzed by two observers (O.M., V.C.). In case of disagreement, the observers reanalyzed the staining results until they reached a consensus.

### Statistical analysis

The statistical analysis was performed using SPSS version 15 software (SPSS Inc, Chicago Illinois, USA). The determination of the optimum cutoff value for survivin immunoexpression in prediction of overall survival was performed by the receiver operating characteristic (ROC) method, along with the determination of sensitivity and specificity of all cutoff values. The chi-square test was used to evaluate the differences in therapy response and survival in relation to clinical and apoptotic parameters. Overall survival (OS) was calculated as the time from establishing the diagnosis to the date of death or last contact. Overall survival was analyzed using the Kaplan–Meier method, and the log-rank test was used to compare the difference in the survival data. A multivariate analysis (Cox’s regression analysis) was performed to examine the effect of presumed prognostic factors on survival. All statistical tests were two-sided, with *p* value ≤0.05.

## Results

### Patients’ characteristics

Clinical data were available for all patients, as summarized in Table 1.

**Table 1:** Clinical data and histological features of 56 DLBCL patients

Age (years)	
Median (IQR)	52.25 (16)
Range	(19–87)
>60	11 (19.64 %)
Gender	
Male/female	32 (57.14 %)/24 (42.86 %)
Stage	
I	1 (1.78 %)
II	15 (26.78 %)
III	21 (37.5 %)
IV	19 (33.92 %)
ECOG	
0	26 (46.43 %)
1	21 (37.50 %)
2	6 (10.71 %)
3	3 (5.35 %)
B symptoms	31 (55.35 %)
IPI	
Low	23 (41.1 %)
Low/intermediate	16 (28.6 %)
High/intermediate	10 (17.9 %)
High	7 (12.5 %)
Bulky disease (≥7 cm)	29 (51.8%)
(≥10 cm)	12 (21.4 %)
Extranodal localization	19 (33.9 %
LDH (>460U/L)	34 (60.7 %)
Median (IQR)	570 (330)
Range	214–2,598
β-2-Microglobulin (mg/L)	
Median(IQR)	4.3 (3.7)
Range	1.18–13.9
CRP (mg/L)	
Median(IQR)	15.5 (35.7)
Range	1.7–285
Lymphocyte count (×10^9^/L)	1.8 (1.2)
Range	0.3–11
Therapy	
R-CHOP/R-EPOCH	51 (91.07 %)/5 (8.93 %)
GBC/non-GBC subtype	19 (51.4 %)/18 (48.6 %)

### Immunohistochemical analysis

**Fig. 1:**
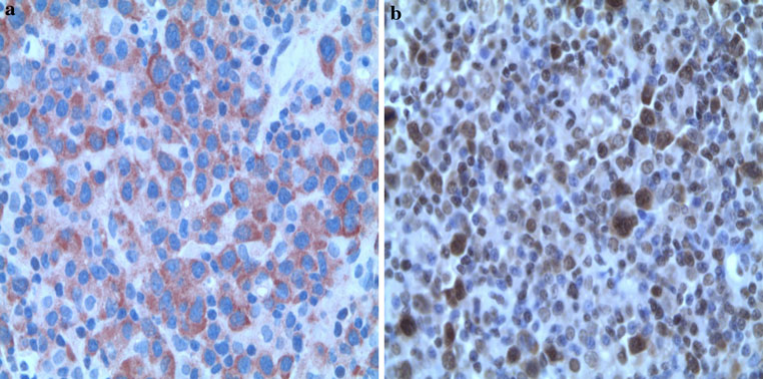
Detection of survivin in biopsy specimens of primary nodal DLBCL—**a** survivin demonstrating cytoplasmic staining, **b** mixed staining (original magnification ×400)

The percentage of positive tumor cells ranged from 1 to 95 % (the mean percentage of positive cells was 36/IQR 57/). According to the results of the ROC method, the optimum cutoff value for survivin immunoexpression was defined as >45 % positive tumor cells. Therefore, survivin immunoexpression was found in 22 (39.28 %) patients and observed as cytoplasmic staining in 15 patients, or as mixed (cytoplasmic and nuclear) staining in 7 patients (Fig. 1).

### Correlation between survivin immunoexpression and subtype and clinical parameters

We noticed a significant difference in survivin immunoexpression between the GCB and the non-GCB subtype of DLBCL (*p* = 0.031), namely survivin positivity was noticed more often in the non-GCB than in the GCB subtype (Table 2). On the contrary, survivin immunoexpression was not in any significant correlation with the analyzed clinical parameters: hemoglobin (*p* = 0.699), IPI (*p* = 0.093), “bulky” disease (*p* = 0.313), extranodal localization (0.397), lymphocyte count (0.327) and Ki-67 immunoexpression (*p* = 0.577).

**Table 2:** Expression of survivin in the GCB and the non-GCB subtype

Expression of survivin (%)	GBC (%)	Non-GBC (%)	*p*
Survivin ≤ 45	15 (65.2)	8 (34.8)	**0.031**
Survivin > 45	4 (28.6)	10 (71.4)	

### Response to therapy

Therapy response was achieved in 45 (80.4 %) patients. We noticed a significant difference in the likelihood of achieving therapy response regarding survivin immunoexpression (*p* = 0.048). However, localization of survivin expression (cytoplasmic vs. mixed) had no influence on therapy response (*p* = 0.98) (Table 3). The relevance of the clinical parameters for therapy response tested by the chi-square test showed a significant difference in the likelihood of achieving therapy response regarding the following clinical parameters: ECOG (*p* = 0.003), β-microglobulin (*p* = 0.03) and clinical stage (*p* = 0.002).

**Table 3:** Therapy response and survival according to expression of survivin

Parameters	Rate of therapy response (%)	*p*	Percent of survived patients (%)	*p*	Percent of relapse (%)	*p*
Survivin (%)						
≤45	31(91.17)	**0.048**	25(73.5)	**0.034**	4(11.84)	**0.131**
>45	14(63.63)		10(45.50)		6(27.3)	
Survivin						
Cytoplasmic	10(66.67)	0.98	7(46.6)	0.21	4(26.66)	0.33
Cytoplasmic + nuclear	5(71.40)		2(42.85)		2(28.57)	

A relapse of the disease was noticed in 10 (17.85 %) patients after a median follow-up of 40 months. There was a difference in the relapse rate related to the immunoexpression of survivin, namely a relapse of the disease appeared in 6 (27.3 %) survivin-positive patients and in 4 (11.1 %) survivin-negative patients, but this difference did not reach statistical significance (*p* = 0.131).

### Overall survival (OS)

**Fig. 2:**
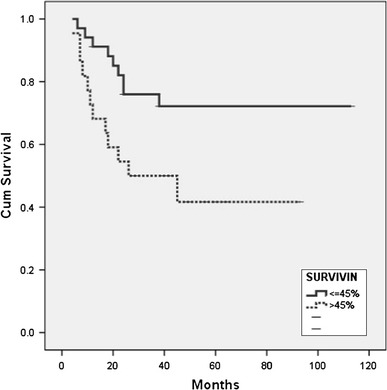
Kaplan–Meyer curve of survival of DLBCL patients according to survivin immunoexpression

**Fig. 3:**
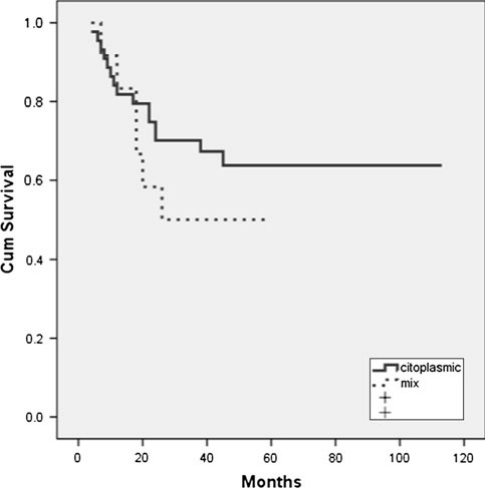
Kaplan–Meyer curve of survival of DLBCL patients according to localization of survivin immunoexpression

The median follow-up period for OS of patients was 40 months (ranging from 2 to 72 months). At the time of the final analysis, 35 (62.5 %) patients were alive and 21 (37.5 %) patients had died. The median survival period of the whole group of analyzed patients was 39 months. A univariate analysis showed that the following clinical parameters were significantly associated with the overall survival rate: ECOG (*p* < 0.001), albumins (*p* = 0.007), ß_2_-microglobulin (*p* = 0.012), extranodal localization (*p* = 0.008), “bulky” disease (*p* = 0.011), clinical stage (*p* = 0.039) and IPI (*p* < 0.001). The immunoexpression of survivin was also significantly associated with the overall survival rate (*p* = 0.034) (Table 3), namely the median survival period of survivin-positive patients was not reached, while the median survival period of survivin-negative patients was 26 months (Fig. 2). There was no statistically significant difference in the survival of the patients regarding localization of survivin expression (cytoplasmic vs. mixed) (*p* = 0.21) (Fig. 3). A multivariate analysis (Cox’s regression model) showed that only IPI is an independent risk factor for the survival of the patients with DLBCL.

## Discussion

Previous studies have shown that deregulation of inhibitory apoptotic proteins is an important factor in the pathogenesis of lymphoma and that such deregulation may be an important cause for chemotherapy resistance and poor prognosis in DLBCL [7–12]. The present study was constructed to clarify the meaning of survivin immunoexpression in patients with nodal DLBCL treated with immunochemotherapy.

Survivin was first identified by Altieri from hybridization screening of a human genomic library with the cDNA of effector cell protease receptor/1(ERP/1) in 1997 [19]. The survivin gene located in 17q25 encodes multiple alternately spliced mRNAs, which appear to be translated into five different splice variants of proteins, the native, full-length anti-apoptotic IAP-survivin, survivin-2B, survivin-∆Ex3, survivin-3B and survivin-2α [20]. It has been demonstrated that some of these isoforms have subcellular localization patterns that could be associated with unique functional properties. Preliminary reports suggest that survivin and survivin Ex3 have anti-apoptotic properties, while survivin 2α attenuates the anti-apoptotic activity of survivin. The function of survivin 2B has not been described. Survivin inhibits apoptosis directly, by binding to and inhibiting the activation of caspase [21], or indirectly, by suppressing the activation of specific proapoptotic factors [22], namely survivin binds and inhibits caspase-9 and Smac/DIABLO function and also binds and stabilizes other IAPs, for example, XIAP, promoting their anti-apoptotic effect (9). In addition to its anti-apoptotic function, survivin plays an essential role in cellular proliferation as an essential component of the chromosome passenger complex [23]. Survivin-∆Ex3 is also responsible for modulating angiogenesis via several mechanisms including cell invasion, migration and Rac/1 activation [20].

Survivin is transiently expressed during embryonic development, but barely detectable in normal, differentiated adult tissue [19, 24]. In contrast, it has been found to be expressed in a wide variety of solid tumors and malignant hematological diseases [19, 25]. In some tumors, a high level of survivin is a risk factor for resistance to chemotherapy and a poor outcome [26, 27]. Overexpression of survivin correlates with reduced remission rates and survival in pediatric patients with acute lymphoblastic leukemia, adult patients with acute myeloid leukemia and adult T cell leukemia, as well as diffuse large B cell lymphoma [9, 28, 29].

In the present study, 39.28 % patients with DLBCL were defined as survivin positive, and this figure is similar to the results of previous studies [7–13]. Our results are also in accordance with the results of most previous studies, which showed that a high level of survivin expression correlates with a reduced remission rate and survival in patients with DLBCL treated with chemotherapy. Since limited data are available on patients treated with immunochemotherapy [13], we showed that overexpression of survivin is in significant correlation with therapy response and the survival of patients with nodal DLBCL treated with immunochemotherapy. In other words, rituximab cannot overcome a negative prognostic impact of survivin overexpression in these patients. We also showed an increased tendency in survivin-positive patients for a relapse of the disease, although this difference was significant only at *p* = 0.131 level.

Literature data about the prognostic significance of subcellular localization of survivin (cytoplasmic or nuclear) are contradictory [20], namely the prognostic significance of cytoplasmic [9, 10, 12], as well as nuclear, positivity [11] has been previously reported. However, it has recently been shown that only cytoplasmic localization correlates with the anti-apoptotic function of survivin and that the sensitivity of cells to chemotherapeutic drugs is even increased when survivin’s localization is restricted to the nucleus [30]. We noticed cytoplasmic staining in most positive cases and mixed (cytoplasmic and nuclear) staining in a smaller number of patients, but a statistically significant difference regarding the remission rate and OS between cytoplasmic and mix (cytoplasmic and nuclear) staining was not found.

We noticed a significant difference in survivin expression between the GCB and the non-GCB type, which means that survivin may contribute to a worse prognosis in non-GCB patients. On the contrary, Watanuki-Miyauchi et al. [31] showed that survivin-positive patients in both subtypes tended to have a poor prognosis.

Interestingly, survivin overexpression was not in any significant correlation with other, well-established clinicopathological prognostic parameters in DLBCL: clinical stage, IPI, “bulky” disease, proliferative activity and extranodal localization. In addition, a multivariate analysis showed that only IPI was an independent prognostic parameter in our study group of patients with DLBCL.

In conclusion, disregulation of survivin in DLBCL is an important step in the pathogenesis of DLBCL. Therefore, survivin represents a very attractive target for new therapies that could lead to further improvement in the treatment for DLBCL. As at least five splice variants of survivin with different functions have been described, further larger studies are required in order to examine the expression of all survivin isoforms and their prognostic significance in DLBCL patients.
